# Combining cognitive bias modification training with motivational support in alcohol dependent outpatients: study protocol for a randomised controlled trial

**DOI:** 10.1186/s13063-015-0576-6

**Published:** 2015-02-26

**Authors:** Marilisa Boffo, Thomas Pronk, Reinout W Wiers, Stefania Mannarini

**Affiliations:** Department of Philosophy, Sociology, Education, and Applied Psychology (FISPPA), University of Padova, via Venezia 8, 35131 Padova, Italy; Department of Psychology, Addiction Development and Psychopathology (ADAPT)-lab, University of Amsterdam, Weesperplein 4, 1018 XA Amsterdam, The Netherlands; Collaborative Antwerp Psychiatric Research Institute (CAPRI), University of Antwerpen, Universiteitsplein 1, 2610 Antwerpen, Belgium; Interdepartmental Centre for Family Research, FISPPA, University of Padova, Piazza Capitaniato 3, 35139 Padova, Italy

**Keywords:** Cognitive Bias Modification, Motivational support, Alcohol, Addiction, Approach bias, Attentional bias, Randomised clinical trial

## Abstract

**Background:**

Addiction research has hypothesised that automatic and reflective cognitive processes play an important role in the onset and maintenance of alcohol (ab)use, wherein automatic reactions to drug-related cues steer the drug user towards consuming before reflective processes can get over and steer towards a different behavioural response. These automatic processes include the tendency to attend and approach alcohol cues. These biases may be trained away from alcohol via computerised cognitive bias modification (CBM). The present protocol describes the design of a double-blind randomised controlled trial (RCT) testing the effectiveness of attentional bias and approach bias re-training with a 2×2 factorial design, alongside a brief motivational support (MS) program.

**Methods/Design:**

Participants (n = 120) are adult alcohol dependent outpatients, recruited from a public health service for addiction in Italy, who have been abstinent for at least two months, and with a main diagnosis of alcohol dependence disorder. Participants are randomly assigned to one of four experimental conditions and complete 11 sessions of training after a baseline assessment. The MS takes place before each training session. Post-intervention and three-month follow-up assessments examine the change in clinical outcome variables and attentional and approach biases (measured with the Visual Probe Task and the Approach-Avoidance Task, respectively). Alcohol approach-avoidance implicit memory associations (measured with the Brief Implicit Association Test) are also evaluated at pre- and post-intervention to explore generalisation effects. Primary outcome measure is relapse rate at follow-up. Secondary outcome measures include change in cognitive biases, in alcohol-related implicit memory associations, and in the clinical variables assessed. An exploratory analysis is also planned to detect interaction effects between the CBM modules and possible moderators (interference control capacity, gender, age, number of previous detoxifications) and mediators (change in cognitive bias) of the primary outcome measure.

**Discussion:**

This RCT is the first to test the effectiveness of a combined CBM intervention alongside motivational support in alcohol-dependent outpatients. The results of this study can be extremely valuable for future research in the optimisation of CBM treatment for alcohol addiction.

**Trial registration:**

Current Controlled Trials ISRCTN01005959 (registration date: 24 October 2013).

**Electronic supplementary material:**

The online version of this article (doi:10.1186/s13063-015-0576-6) contains supplementary material, which is available to authorized users.

## Background

People with an addiction disorder often describe their substance (ab)use as a somewhat ‘unconscious’ decision, something that happens ‘by chance’ and without any intentional planning or awareness. However, many drug users are conscious of the detrimental effects of substance abuse and seek treatment to abstain from consuming, citing rational considerations such as the costs of continued substance use outweighing the benefits. Nevertheless, the risk for lapse and relapse remains extremely high. This paradoxical and deleterious pattern of behaviour encouraged research into the mechanisms underlying drug-seeking behaviour even when explicit motivations to quit are present.

It has been long known that behaviour is not only driven by rational and conscious processes, but also driven by mechanisms that go beyond intentionality (see Hofmann *et al*. [1]). Recently, this realisation has gained relevance in addiction research, resulting in efforts examining the role played by relatively automatic processes in the onset and maintenance of addictive behaviours [2-7]. At a descriptive level, dual-process models of addiction point towards two qualitatively different classes of information processing mechanisms underlying behaviour, namely:

- Automatic processes, which are initial, fast, associative and impulsive processes evoked by drug-related stimuli, that operate at early stages of response selection through associative links and emotional and motivational associations [7]. When an automatic process leads to impulsive responses or task interference conflicting with explicit task goals, as may well be the case in drug addiction, they are frequently referred to as *cognitive biases*. Cognitive biases are based on associative learning processes and prior reinforcement and are by nature difficult to voluntarily change and control. Examples of cognitive biases are selective attention (for example, alcohol dependents’ attention is usually automatically and selectively drawn by alcohol-related cues), substance-related automatic associations in memory (for example, alcohol being repeatedly and automatically associated to positive or negative feelings) and action tendencies towards the substance (for example, an automatic tendency to approach alcohol cues) [8].
- Reflective processes, which are slower, relatively controlled propositional processes that include ‘rational’ decision-making and emotion regulation. Reflective processes continuously update and integrate initial inputs into more coherent and complex mental representations in order to optimise the selection of the final behavioural outcome [7,9,10]. To achieve this, top-down control is deployed over impulses to integrate them with explicit motives, beliefs and expectancies about the long-term behavioural outcomes and goals [9,10].

The two classes of processes depend on each other and synergistically interact in determining behaviour. That means there is no process that is purely reflective or automatic, though the degree to which each is a more prominent determinant can be distinguished on the basis of their *latency*. For instance, an initial automatic reaction to a positive incentive stimulus can be evoked (bottom-up input), such as an approach response to a glass of wine. After that, the initial reaction can (or cannot) progressively unfold into the action of grabbing the glass and drinking it under the gradual influence of conscious goals and motivation (top-down regulation), such as the explicit goal of alcohol abstinence to re-gain the lost job position [7,9,10].

According to this dual-process perspective, the ‘addiction paradox’ can result from an imbalance between strengthened automatic reactions to substance-related cues that have acquired a high incentive salience after repeated consumption, while weakened reflective processes and cognitive control are obstructed before they can determine the optimal behavioural response [7,8,11]. This imbalance between the operating processes makes the individual more at risk of being triggered by drug cues, thus being automatically prompted to consume the drug, while any deliberate decision to abstain is too late to prevent the individual from consuming [3,7,8].

Since both strengthened automatic reactions to alcohol cues and weakened reflective processes may be interwoven in alcohol-related problems, they both may be targeted in interventions. Reflective processes are usually the focal target of standard treatment interventions, such as cognitive behavioural therapy, counselling and motivational interviewing. A common approach across such interventions consists of the therapist and patient making an explicit analysis of the pros and cons of the patient’s alcohol use, of related motives and expectancies and of strategies and long-term goals to enact deliberate control.

More recently, a new family of interventions, collectively called Cognitive Bias Modification (CBM), has been developed to tackle prepotent, drug-evoked automatic processes involved in addiction [8,12,13]. CBM paradigms are computerised tasks aimed at training alternative responses to a drug-related stimulus in order to adjust the biases that underlie the breakdown in controlled processes. Typically, they are modified versions of assessment tasks, such as the Approach-Avoidance Task (AAT) [14,15], with a built-in contingency that recasts them to re-training paradigm.

The first clinical applications of CBM paradigms as an add-on to standard interventions such as cognitive behavioural therapy have shown promising results [16-19]. Two randomised controlled trials (RCTs) with German alcohol-dependent inpatients did demonstrate that an approach bias for alcohol stimuli could be re-trained, with a generalisation of the training effects beyond the experimental procedure context [18,19]. Moreover, participants in the training condition had a lower rate of relapsing within one year after discharge, after controlling for gender in Wiers *et al*. [18], and for age and number of previous detoxifications in Eberl *et al*. [19]. A study with Dutch alcohol-dependent inpatients showed the success of an attentional bias re-training in increasing the ability to disengage from alcohol stimuli, with a generalisation to untrained, new stimuli and a significantly longer time until relapse for the experimental group in the follow-up period [17].

These results could suggest that computerised CBM has the potential to be an effective intervention that can be easily incorporated into standard health care practices, alongside standard treatments. As a next step, it is important that the findings are replicated and extended across various patient categories and delivery modes, for example by combining different CBM paradigms. To the best of our knowledge, there is only one ongoing RCT testing a completely online CBM intervention combining the CBM training for alcohol approach bias, attentional bias and automatic memory associations among problem drinkers [20]. However, no studies have yet investigated the effectiveness of a combined CBM intervention with alcohol-dependent outpatients. Unlike residential treatment programs, the outpatient treatment regimen does not provide patients with the safe, secure and structured environment that isolates them from negatively influencing factors and tempting conditions. Patients return to their own daily environments after outpatient alcoholism treatment, and must voluntarily abstain from alcohol use, which requires a greater amount of diligence and efforts. Therefore, this group of patients is also likely to receive potential benefits from this type of interventions.

Finally, inspired by the dual-process account of addictive behaviours previously presented, which emphasises the interaction between bottom-up and top-down processes [7-10], this study is the first to include a targeted motivational support (MS) intervention alongside the combined CBM training, in order to integrate long-term goals with the training and tackle deliberate and conscious explicit motivational processes related to the patients’ experience of re-training their alcohol-triggered automatic responses. The main goal of combining CBM with a motivational approach is to enhance motivation to train and harness the individual’s capacity for change [21]. At the start of each training session a brief MS interview is then included on top of the CBM training, with a threefold objective:

- To stimulate and bolster the explicit motivation and reasons for doing the training (goal maintenance, recall of long-term expected outcomes and top-down influence), which can act as a fuel to develop and exert control to overcome the appetitive motivational and automatic reactions towards alcohol [7,8];
- To provide continual feedback about the participant’s subjective performance on the training (incentive value);
- To promote the acceptability of the training ([7,8]) by increasing engagement to intervention (fostering commitment to change).

### The present study: aims and hypotheses

The aim of the current study is to investigate the effectiveness of combining computerised alcohol attentional bias and approach bias CBM trainings in adult alcohol-dependent outpatients alongside a motivational approach to sustain the training progression. The primary goal is testing the main and added effects of the CBM interventions on remission progress from alcohol addiction immediately after the intervention and three months later, with changes in the number of lapse or relapse episodes as primary outcome measure. It is expected that, for each of the two CBM training modules, participants in the active training condition will show a lower percentage of lapse or relapse than participants in the placebo condition [16-19].

Secondary outcome measures include changes in alcohol-related cognitive biases following the combined CBM intervention. Interaction effects between the CBM modules are explored, as well as the additive effect of exposure to the combination of the two CBM trainings. It is expected that each CBM paradigm will decrease or reverse the targeted bias and that these changes might mediate the effects on the clinical outcome (see Eberl *et al*. [19]). Further, it is expected that joint exposure to both active CBM re-trainings will have a greater beneficial effect than each of the active CBM re-trainings separately.

Additionally, we study whether CBM effectiveness is moderated by participant characteristics, namely interference control capacity and strength of the cognitive biases at pre-test. Individual differences in executive functioning can moderate whether the initial automatic reaction towards a glass of wine can be effectively and easily controlled or not. It has been suggested that prolonged alcohol use can negatively affect the deployment of reflective processes (such as interference control and working memory capacity [22,23]), thereby reducing the cognitive capacity available for controlling automatic impulses [8]. Hence, alcohol dependents with a relatively weak ability to inhibit a prepotent approach response (low interference control capacity) may find it more difficult to remain abstinent than those with a relatively strong ability to inhibit a pre-potent response. CBM interventions could be particularly beneficial for those who present relatively poor interference control capacity and/or strong automatic reactions demanding great control efforts. Therefore, in line with dual-process models of addiction and prior findings [8,18,19,24-26], it is expected that participants with strong automatic biases and/or weak interference control will benefit more from the CBM intervention than participants with weaker biases and/or stronger executive functions.

Finally, the effect of several independent clinical variables (such as age, gender and number of previous detoxifications) on the primary and secondary clinical outcomes will be explored, in particular the type of treatments participants are receiving alongside the CBM, such as medication intake and/or other psychotherapeutic interventions.

## Methods/Design

### Trial design

The present study is a phase II, double-blind, parallel group RCT. Following the design of previous CBM studies [18,19], participants complete 11 sessions of either the active or placebo version of both attentional and approach bias training modules. The experimental intervention has a 2×2 factorial design, which combines the active and placebo training modules into four experimental conditions: one double active training experimental condition, two experimental groups receiving one active training and one placebo training and one double placebo training control group. According to the experimental design, the probability of receiving at least one active CBM intervention reaches 75%. The placebo version of each training module consists of a continuous assessment, in which half of the trials train the cognitive biases away towards alcohol and the other half towards soda (active placebo). The manipulation of the stimulus-response contingency in each task allows for a controlled and clean comparison between experimental conditions while retaining the same tasks, stimuli and instructions. Also, Wiers *et al*. [18] found no differences between continuous assessment and no-training placebo conditions, which suggested that either type of placebo could be used.

Participants complete a total of 14 sessions: two baseline assessment sessions, 11 training sessions, one post-intervention assessment session and one three-month follow-up assessment session. Participants complete the training sessions with a between-session time interval of a maximum of five days. The post-intervention assessment takes place between the 10th and 11th training session during a ‘masked’ session (participants do not know they are completing the post-intervention evaluation). That is to avoid possible negative feelings related to the final ‘evaluation’ of the intervention and minimise self-presentation biases and/or preparatory strategies.

**Figure 1:**
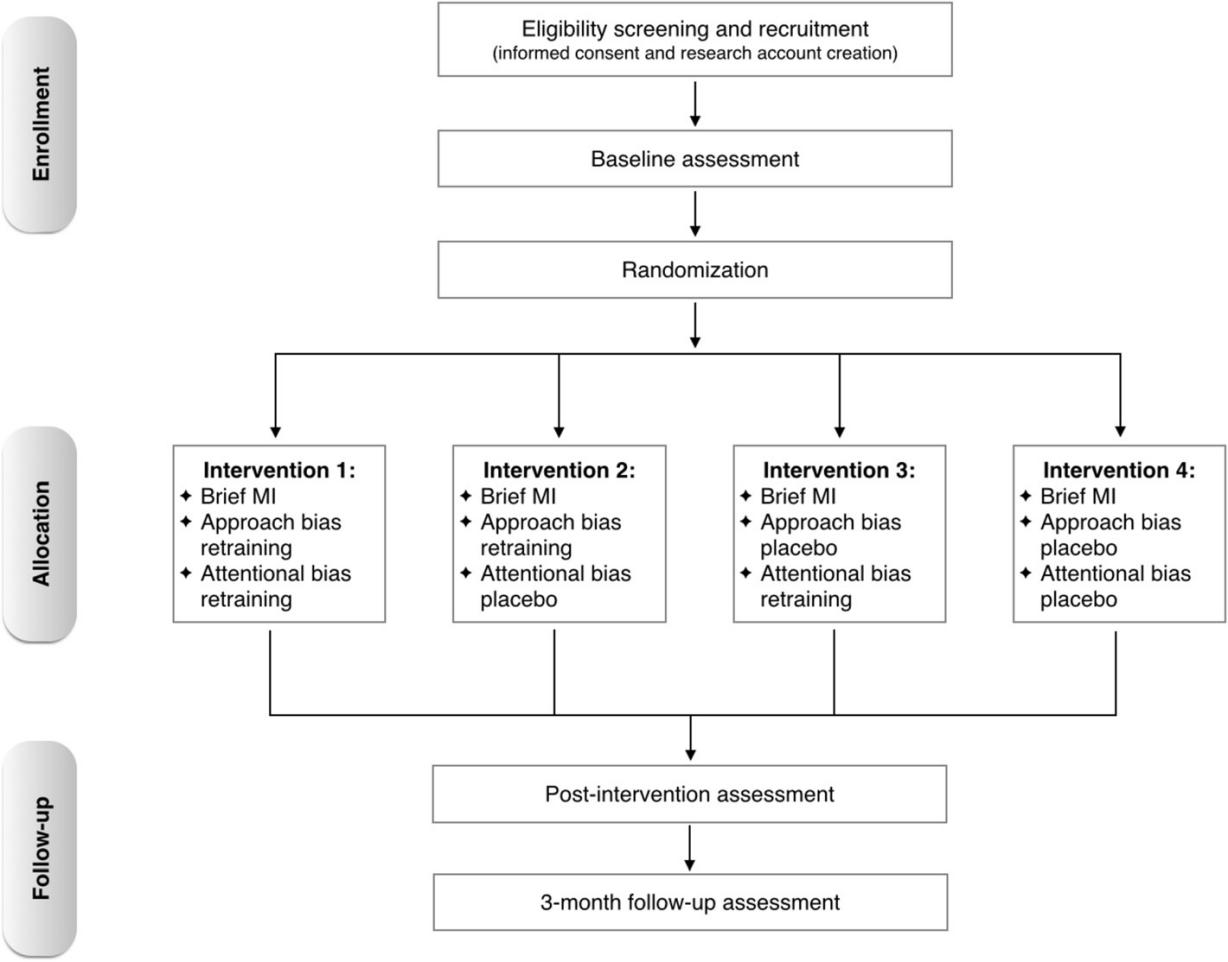
**Participant flow diagram.**

Participant flowchart as per Consolidated Standards of Reporting Trials (CONSORT) statement [27] is presented in Figure 1. The study was approved by the Ethics Committee of the School of Psychology of the University of Padova (March 2013; Protocol number: 1242) and registered at Current Control Trials (identifier: ISRCTN01005959).

### Participants and procedure

Participants are adult outpatients with a main diagnosis of alcohol addiction disorder, recruited from the public health addiction service (Servizio per le Dipendenze, ULSS10) of San Donà di Piave, Italy. The targeted number of recruited participants is 120, to be equally balanced across the four experimental conditions.

Participants are screened for eligibility according to the following criteria:

1. Inclusion criteria: adult outpatients with primary diagnosis of alcohol addiction disorder according to the diagnostic criteria of the Diagnostic and Statistical Manual of Mental Disorders (4th Edition, Text Revision) and alcohol abstinence for at least two months.
2. Exclusion criteria: neurocognitive problems, visual or hand-motoric handicaps, severe neurological disorders (such as Korsakoff syndrome), comorbidity with psychotic disorders or low fluency in the Italian language.

Participants are recruited by clinicians according to the inclusion and exclusion criteria and invited to participate in the study. At invitation, clinicians provide the patients with a brief introduction to the study by explaining that addiction disorders are partly due to uncontrolled and automatic processes which can substantially increase the risk for relapse, and that the main objective of the research is to test the effectiveness of new computerised treatment interventions, which can help the patient in gaining and increasing a better control over these underlying mechanisms. The clinician will also supervise patients’ activity and progress along both the patient’s standard treatment and the experimental intervention.

After the patients are informed about the research objectives and the norms for confidential data treatment and for participating in the study, interested participants undersign a written informed consent form and create their research account at the training website.

Participants will be excluded from the study if they do not complete the baseline assessment or if they disclose the intention to discontinue the study. All participants will continue their treatment as usual in the public addiction service. Upon completion of the three-month follow-up assessment, they have the opportunity to attend the double active CBM training if they wish. The percentage of participants that enter the booster training is registered.

### Randomisation

Participants are automatically randomised at the pre-intervention stage across the four experimental conditions, stratified by gender and category of medication intake, as specified here under:

1. CategoryA (alcohol agonists and antagonists): Disulfiram, Naltrexone, Acamprosate and Gamma-hydroxybutyrate;
2. Category B (psychoactive medication such as anxiolytic, antidepressant and neuroleptic medications);
3. Category C (other medications).

Participants are randomly allocated to one of the conditions to which the fewest participants of their gender and medication category have been so far assigned.

### Blinding

The trial has a double-blind design; hence, both participants and researchers do not know which experimental condition participants are assigned to. Participants create their personal research account at registration in the trial online platform and login to their personal account at each session. Participants are anonymised via the assignment of a user ID number. In order to reduce potential biases, participants complete online assessment measures, tasks and re-training interventions on their own at the computer, with the support of the researcher when requested. Furthermore, to keep participants blind to which intervention they receive they are required to respond to an irrelevant feature of the pictures in both CBM modules (orientation of the picture), instead of reacting to the content of the picture (alcoholic or non-alcoholic drinks) [28] (for a discussion about the difference between relevant- and irrelevant-feature tasks, see Wiers *et al*. and Field *et al*. [29,30]). Participants’ awareness about which experimental condition they are assigned to is assessed during the follow-up assessment.

### Intervention

Each CBM session starts with the brief MS interview (about 15 minutes), after which the motivation for training is briefly assessed, and proceeds with the two training modules (about 15 minutes each).

### Brief motivational support interview

According to the Trans-theoretical Model of Behaviour Change [31,32], which assumes that the changing process in health behaviour treatment is composed of six stages of readiness, from the pre-contemplation (avoidance and denial of a problem) to the maintenance (maintaining the successful changes into the daily life) stage, the participants of the present study are supposed to be in the fourth action stage of change, as assessed at baseline with the alcohol version of the motivation to treatment questionnaire (MAC-2A [33]). This stage is characterised by the pursuit of concrete decisions and behaviours aimed to tackle the addiction problem and to change the status quo, and by the establishment of an intentional commitment to the treatment process.

At the beginning of each training session, participants take part in a brief interview with a trained researcher (about 15 minutes), aimed at introducing the participant to the upcoming experimental session. The protocol of MS here devised has been partially inspired by the principles of the Motivational Interviewing approach [21], developed in the wake of the Trans-theoretical Model of Behaviour Change. Experimenters conducting the MS intervention are firstly trained with a professional in motivational interviewing, and later can practice the MS protocol during several role-plays with clinicians working in the addiction service. The interviewers regularly take part in supervision meetings with the same clinicians to discuss any issue related to the MS interviews.

The main objective of the MS during the action stage is to support the participants’ changes and progress reached so far in a constructive and open-minded way, by explicitly sustaining the efforts they are making to concretely face their addiction problems. In this context the efforts the participants are making include participation in the training sessions. The targeted MS then serves the purpose of preparing and introducing the participant to the upcoming experimental session, by rehearsing the objectives of the training, empowering the motivation to engage in the therapeutic process and sustaining the patient’s attention to the potential benefits of the CBM trainings. Namely, the strategies adopted in this phase are: reviewing progress, renewing motivation and redoing commitment [34].

In particular, each session of MS should cover the following topics:

1. Review of the previous training session (except the first training session) and feedback to co-structure a positive framed feedback on the progress so far.
2. Renewal and support of the motivation and compliance to the experimental intervention. According to the participant’s report of the previous session, the interview proceeds by shifting the attention to the motives that led the participants to start the change process, to support and/or empower them and remind them of the objectives of this training. The joint rehearsing of the motivations that brought the patient to undergo a treatment intervention (such as ‘I don’t want my loved ones to be ashamed of me’, ‘I don’t want my children to grow up with an alcohol addicted father/mother’, ‘I messed up my life because of the drinking’ and ‘I don’t want to feel like a loser anymore’) should work as a fuel for continuing the intervention (long-term goals) and prepare the ‘field’ for the engagement of top-down control processes.
3. Empowerment of self-efficacy and affirmation of the current changing progresses. The training interventions are one of the concrete actions pursued by the participant to deal with the alcohol abuse. It is then important to explicitly acknowledge the progress (precision, constancy and commitment to the tasks) and to reinforce it (for example, by stressing that the practice effect can be a sign of individual efficacy in performing the tasks and increased control over the performance).
4. Normalisation of any difficulty encountered in the training execution and reaffirmation of commitment (for example, by using the metaphor of gym training). Difficulty and boredom are common experiences that are intrinsically part of these kinds of interventions. In particular, the repetition of a certain behavioural pattern is a key component when the objective is to strengthen ability, or a muscle for example, or to increase the expertise and control in some life domains, such as a job activity. This strategy is functional to the reinforcement of the participant’s sense of autonomy and ability to pursue self-chosen goals and plans.
5. Collaborative negotiation of the incoming session proximal goals. What are the expectations for the next session? What is the goal the participant would like to reach? For example, the proximal goal of increasing the response speed or reducing the number of errors sounds like a challenge for some participants and consequently stimulates their active involvement in the task.

Generally speaking, the interview is conducted with a specific, empathic and non-judgemental interviewing style and should be based on a concrete level of interaction, client-centred and focused on the introduction and practical discussion of the training sessions. The discussion of personal feelings and experiences related to the individual case study and relevant to the patient’s therapeutic path should be acknowledged but still re-addressed to the standard psychotherapeutic setting. According to the Trans-theoretical Model of Behaviour Change perspective, the change processes involved in the MS here devised deal with behavioural processes, namely, reinforcement of self-efficacy and managing strategies, stimuli control and relationship support, leaving the in-depth involvement of the experiential processes to the individual clinical setting.

Neither the participant nor the researcher knows to which experimental condition the participant has been assigned. If participants enquiry about their intervention condition the researchers will honestly disclose their own unawareness about participants’ allocation.

### Cognitive Bias Modification interventions

Each CBM training session consists of two modules: the attentional bias re-training and the approach bias re-training. Each task, both in the active and placebo version, consists of three blocks: a brief practice block, a mini-assessment block and a training block. The practice block presents neutral stimuli (grey geometrical pictures) to practice the task. The mini-assessment block serves the purpose of measuring the strength of the bias at the start of every session and tracking any change in the cognitive bias as a result of the CBM training.

In both tasks, each trial starts with a fixation cross in the middle of the screen for a duration randomly drawn from a *U*[500, 1,000] distribution. This setting was designed to make the task less boring, to keep participants’ attention focused and to avoid anticipatory responses. Whenever a wrong response is given, a red cross appears on the screen to allow for correction.

The two tasks were designed to be as similar as possible (same stimuli and number of trials) to avoid any confounding effect on participants’ performance. Their order of presentation is counterbalanced between subjects and fixed within subjects across all measurement time-points.

#### Attentional bias retraining

**Figure 2:**
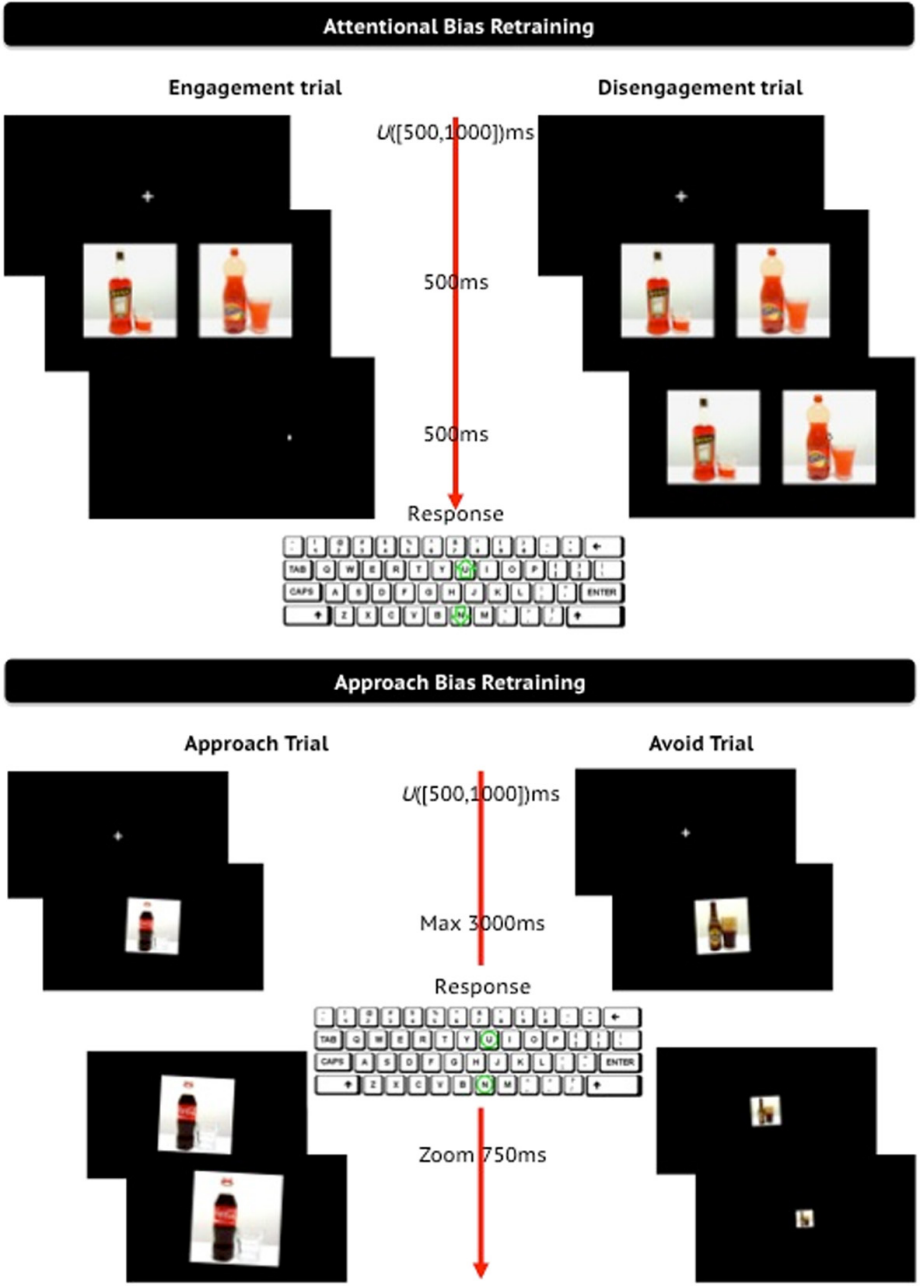
**Example of trials in the attentional bias re-training (upper half) and in the approach bias re-training (bottom half).**

Attentional bias is assessed and trained through a modified version of the Visual Probe Task (VPT) [12,13,20], which is a computerised speeded reaction-time task in which participants are asked to respond to probes located in two different positions on the computer screen (irrelevant-feature implicit measure [30]). During the task, a picture of an alcoholic drink and a picture of a non-alcoholic drink are presented next to each other on the screen for 500 ms. After the stimuli presentation, a small arrow (8.3% of the width and height of the picture) pointing upwards or downwards is presented in either of two trial formats: in half of the trials it replaces one of the two pictures (after format), measuring speeded detection of alcohol-related stimuli (attention engagement), and in the other half it is positioned on top of one of the pictures (on-top format), measuring the relative difficulty to disengage from alcohol-related stimuli (attention disengagement). Participants are instructed to respond as fast as possible to the direction of the arrow by pressing the corresponding key on the keyboard (U and N) (for an example of a trial, see Figure 2). Response window is set to 4,000 ms; in cases of no response, the trial is restarted after repeating the task instructions.

In the VPT assessment version and in the mini-assessment block during training, the arrow replaces the picture of alcoholics (alcohol trials) and non-alcoholics (non-alcohol trials) equally often. Attentional bias is computed by subtracting the median response time (RT) on alcohol trials from the median RT on non-alcohol trials, separately for after and on-top trial formats. In the training block, participants in the experimental condition are trained to direct their attention away from alcoholic drinks towards non-alcoholic drinks by exposing them only to non-alcohol trials, whereas participants in the placebo condition are presented with 50% alcohol and 50% non-alcohol trials (as in the VPT assessment version and in the mini-assessment blocks).

Stimuli are pairs of matched alcohol- and non-alcohol pictures, which are counterbalanced with a 2×2×2 design for assessment and placebo training (stimuli presented on the left and on the right, formats of arrow presentation and arrow location on the alcohol or non-alcohol picture), and with a 2×2 design for active training (stimuli presented on the left and on the right and formats of arrow presentation). The probe direction is set randomly upwards or downwards with the restriction that up and down appears equally often.

#### Approach bias re-training

Alcohol automatic approach tendencies are assessed and trained with the Approach-Avoidance Task (AAT) [14,15,20,28], which is a computerised speeded reaction-time task in which participants are asked to react to the presentation format of the stimulus and ignore the stimulus content (irrelevant-feature implicit measure [30]).

In this task, a picture of an alcoholic or non-alcoholic drink is presented in the centre of the screen. The picture is tilted to the left or right by three degrees. Participants are instructed to respond to the tilt direction of the picture by pushing pictures tilted to the left away from them, and pulling pictures tilted to the right towards them, by pressing and holding two keys (U and N) on the keyboard. The combination of picture tilt direction and response (left/push and right/pull versus left/pull and right/push) is counterbalanced across participants. Participants’ response is accompanied by a zooming effect, which increases picture size in the pulling closer response and decreases it in the pushing away response, mimicking actual approach and avoidance (for an example of a trial, see Figure 2).

In the AAT assessment version and in the mini-assessment block during training, pictures of alcoholics and non-alcoholics are presented equally often in both formats. The approach bias score is computed following the conventional D-score algorithm generally used in the Implicit Association Test [35], and adapted by Wiers *et al*. to the AAT [18], which standardises the difference in RTs by dividing the difference by the individual standard deviation (SD) of RTs. An approach bias D-score is computed for alcohol ((alcohol/push − alcohol/pull)/SD(alcohol)) and for non-alcohol trials ((non-alcohol/push − non-alcohol/pull)/SD/non-alcohol)). Positive scores indicate an approach tendency, negative ones indicate an avoidance tendency.

In the training block, participants in the experimental condition are trained to avoid alcohol by exposing them only to alcohol/push and non-alcohol/pull trials, whereas for participants in the placebo condition alcoholic and non-alcoholic beverages are presented equally often in both formats (as in the AAT assessment version and in the mini-assessment blocks).

Stimuli are pairs of matched alcohol and non-alcohol pictures, which are counterbalanced for presentation format only for assessment. Stimuli stay on screen for 3,000 ms; in cases of no response the trial is restarted after repeating the instructions.

#### Tasks stimuli

The current beverage picture set is an Italian adoption of the Amsterdam Beverage Picture Set (ABPS; Pronk T, Van Deursen DS, Beraha EM, Larsen H, Wiers RW. Validation of the Amsterdam Beverage Picture Set: a controlled picture set for cognitive bias measurement and modification paradigms. Forthcoming). As such, half of the pictures are passive pictures that display beverages in front of a white background, the other half being active pictures that display beverages being held, opened, drunk or served by a model in front of a white background. Prior research indicated that for heavy drinkers, alcohol displayed in a social setting induced stronger self-reported craving [36] and stronger emotional responses [37] than alcohol in front of a white background. This may be due to alcohol in a social setting being a more naturalistic cue than the alcohol without any context. In contrast, pictures without context did induce a stronger attentional bias [38] than social pictures. This may be due to fast mental processes such as cognitive biases being most effectively triggered by simple stimuli that solely feature a stand-alone alcoholic or non-alcoholic beverage. The pictures used in the current study were designed to be relatively simple, but nevertheless feature beverages both with (active) and without (passive) drinking contexts. This distinction is aimed at keeping pictures relatively simple, while allowing for a sufficiently varied stimulus set.

The stimulus set features 144 pairs of alcohol and non-alcohol pictures matched by structural, visual and pictorial features and photographed in both passive (beverage only) and active (presence of a human in interaction with the drink) contexts. Alcohol pictures depict highly popular and recognisable Italian brands of wine, beer and spirits. A common non-alcoholic drink was selected for each alcoholic beverage by matching, as much as possible, the type of packaging (bottle, can, jar or carton), package size and colour.

Drinks were then photographed in a neutral setting (windowless room with a table on a white background, full illumination on the centre of the table, various glasses for the different drinks, a tray and a bottle opener) and according to the following criteria: drinks in the foreground of the picture, consistent framing to shoot pictures from the same angle and use of a standard digital camera.

Drinks were photographed in six scenarios, three for each context (passive: open beverage only, open beverage with empty glass and open beverage with full glass; active: woman serving the open drink on a tray, woman or man opening the drink and woman or man drinking). In woman or man active scenarios, alcoholic drinks, as well as their matched non-alcoholic drinks, were counterbalanced for drink category (wine, beer and spirits) and gender; whereas in the three passive scenarios each picture was shot in each scenario.

Stimuli were processed in Adobe Photoshop CS6 (Adobe Systems Incorporated, San Jose, California, USA) to adjust for size, exposure, brightness, contrast and to correct minor image imperfections (see Additional file 1: Table S1 for some examples of stimuli). Stimuli are randomised with 50/50 proportion of passive and active pictures in each task. In each training session untrained and already trained pictures are presented. At the pre- and post-intervention assessment session the VPT and AAT use different untrained stimuli. The follow-up measurement session is equal to the post-intervention session; the same stimuli are used.

### Measures

An overview of all measurement instruments along the RCT measurement time-points is presented in Table 1.

**Table 1:** **Measurement instruments: purpose, measures and time points**

**Purpose**	**Measures**	**Baseline**	**Training**	**Post-intervention**	**Follow-up**
Cognitive bias assessment	VPT AAT	✓	✓	✓	✓
Generalisation of training effects	BIAT	✓		✓	
Executive function	Stroop task	✓		✓	
Baseline measures	Demographics Case history details RSES	✓			
Primary outcome measure	Clinical status (lapse/relapse)	✓		✓	✓
Secondary outcome measures	AUDIT^a^ CORE Alcohol/Drug use^a^ OCDS STAI-Y BDI-II	✓		✓	✓
Motivation to treatment	MAC2-A	✓		✓	
	Motivation to training		✓		

^a^At baseline, post-intervention and follow-up measurement sessions, the questionnaire refers to the last 12, 1, and 3 months, respectively.

VPT: Visual Probe Task; AAT: Approach-Avoidance Task, BIAT: Brief Implicit Association Task, RSES: Rosenberg Self-esteem Scale, AUDIT: Alcohol Use Disorder Identification Test, OCDS: Obsessive-Compulsive Drinking Scale, STAI-Y: State-Trait Anxiety Inventory – Form Y, BDI-II: Beck Depression Inventory-II, MAC2-A: Motivation to Change-Alcohol version.

### Baseline measures

Sociodemographic information (gender, birthdate, annual income and educational level) and clinical case history details (duration of alcohol addiction, previous detoxifications and treatments, duration of current abstinence and medication intake) are collected during participants’ research registration.

At the first baseline assessment session, other substance use (integration of CORE Alcohol and Drug Abuse Survey (CORE Institute) and the Italian Population Survey on Alcohol and other Drugs questionnaire (National observatory for Drug Use)), self-esteem (Rosenberg Self-Esteem Scale, RSES) [39,40], anxiety (State-Trait Anxiety Inventory-Y, STAI-Y) [41] and depressive symptoms (Beck Depression Inventory-II, BDI-II) [42] are evaluated. After the questionnaires, participants are assessed for baseline alcohol approach bias (VPT) and attentional bias (AAT).

At the second baseline assessment session, alcohol abuse (Alcohol Use Disorders Identification Test (AUDIT)) [43], craving (Obsessive-Compulsive Drinking Scale, OCDS) [44] and motivation to treatment (MAC2-A) [33] are evaluated.

After the questionnaires, a computerised version of the classical Stroop task [45,46] is used to assess interference control capacity [18,19,26]. In this task, participants have to classify words and symbols according to their ink colour and ignore the content. The task starts with a practice block, in which participants have to learn the correct key-colour combination (only neutral and incongruent trials are presented). The second block consists of a second practice block task with grey key reminders on the bottom of the screen. The third block is a test block composed of 112 trials in which the key reminders disappear and 16 neutral trials (such as symbols like #### in blue ink colour), 48 congruent trials (such as the word ‘red’ in red ink colour) and 48 incongruent trials (such as the word ‘red’ in yellow ink colour) are presented.

The second assessment session ends with a Brief Implicit Association Task (BIAT) [47] measuring the strength of approach and avoidance associations with alcohol [18,19]. In the BIAT, participants are required to choose whether word stimuli presented in the centre of the screen belongs to one of two focal categories on top of the screen or not, by pressing the ‘yes’ and ‘no’ corresponding keys (E and I). In the first block (16 trials) participants practice the task by classifying words for alcoholic beverages (wine, beer, vodka and rum), non-alcoholic beverages (pepsi, milk, water and tea), mammals (horse, sheep, cat and elephant) and birds (swallow, eagle, hawk and pigeon), as belonging to alcohol or mammals (focal categories) or not (‘anything else’ category). In the subsequent four blocks (20 trials each), the alcohol focal category is alternatively paired with approach (block two and four) or avoid (block three and five) attribute category. Test attribute stimuli for approach (grab, approach, closeness and touch) and avoidance (flee, push, avoidance and elude) have been adapted from Wiers *et al*. [18] and Ostafin and Palfai [48]. The order of the combined blocks for the alcohol/[attribute category] pairings within the BIAT and the contingency between the response and the assigned key (E and I) are counterbalanced across participants. The outcome measure is computed as the standardised difference in latencies between the different combined blocks (modified D-score algorithm [49]). As a control measure, participants subsequently rated BIAT stimuli on valence with a visual analogue scale from 0 (extremely negative) to 10 (extremely positive).

### Outcome measures

The main outcome measure is the occurrence or not of lapse and/or relapse episode(s) during the three months after the intervention (follow-up assessment). The treatment status (any medication intake and other form of therapeutic intervention) at the follow-up assessment is also considered in comparison to the baseline status.

Secondary outcome measures include changes in the automatic cognitive biases as assessed with the VPT and AAT at post-intervention and follow-up assessment sessions. At the follow-up assessment, VPT and AAT present the same stimuli of the post-intervention session, to check for the duration of the training effects.

Generalisation effects of the two CBM modules to other measures are assessed at post-intervention with the BIAT [20-22]. Other secondary outcome measures (assessed at each measurement point) also include other substance abuse (CORE questionnaire), alcohol-related problems (AUDIT), craving (OCDS), anxiety (STAI-Y), depression symptoms (BDI-II) and motivation to treatment (MAC2-A; measured at pre- and post-intervention only).

Intervention credibility and expectancies are also assessed with the Credibility/Expectancy Questionnaire [50] to evaluate participants’ general experience with the study.

### Data analysis

Complete analyses will be conducted in agreement with Intention-To-Treat principle [26]. Missing data points will be handled with multiple imputation.

To answer to the main research question, a multiple logistic regression will be used to estimate how much the main and combined exposure to the two CBM modules affects the occurrence of lapse and/or relapse episodes at the three-month follow-up assessment.

The secondary research question, whether participants approach and attentional biases do change after the CBM intervention, is addressed via a 3×2×3 repeated-measures mixed ANOVA with the AAT and VPT scores as dependent variables, time (baseline, post-intervention and follow-up) as repeated measures within-subject factor, drink type (alcoholic and non-alcoholic) or attentional process (engagement and disengagement) as within-subject factor, and CBM condition (re-training or placebo) as between-subject factor. The same analysis applies to the continuous secondary outcome measures administered at each time point.

The third research question of the present study is framed within a ‘what works best for whom?’ approach [8] and explores the moderating and mediating roles of individual differences variables on the clinical outcome by means of a moderated mediation analysis [51]. More precisely, baseline strength of the cognitive biases and interference control capacity (Stroop task), as well as age, gender and previous detoxifications, are hypothesised to moderate the change in cognitive bias after the CBM intervention, which is further hypothesised to eventually mediate the change in the clinical outcome variable (see Eberl *et al*. [19]).

Finally, participants’ motivation to change (MAC2-A) at pre- and post-intervention will be compared via a paired t-test. Via a repeated-measures ANOVAs we will explore whether change in participants’ motivation to carry on with the training after the brief MS interviews affects attrition and training outcome.

### Sample size

The sample size is limited to 120 recruited participants due to constraints in the ratio between the number of potential participants fully meeting the eligibility criteria and available to participate in the study, out of the total number of alcohol-dependent outpatients currently in charge in the public service at the time of recruitment (3:10). Therefore, a realistic estimate of 120 participants (n = 30 per experimental condition) was considered achievable for a phase II RCT study, and further supported by a minimum number of cases identification analysis.

An operational guideline to identify the minimum number of cases to include in the study has been suggested by the Peduzzi *et al*. [52]. For *k* independent variables and *p* as the smallest of the proportions of negative or positive cases in the population, then the minimum number of participants is 10*k/p. Based on three previous CBM studies with alcohol dependent clinical populations, the average proportions of occurrence or absence of relapse event(s) at follow-up are about 0.53 and 0.47 after an approach bias or control re-training, respectively [18,19]; and about 0.40 and 0.28 after an attentional bias or control re-training, respectively [17]. The proportions were computed out of the total number of enrolled participants.

By taking the minimum among the four proportion values and setting *k* = 2 (two independent variables: attentional bias CBM re-training or placebo and approach bias CBM re-training or placebo), the minimum number of cases to be included in the present study is 10*2/0.28 ≅ 71. According to this approach, the proposed sample size is more than adequate to answer to the primary research question of the present study and should also allow for high attrition rates. It is worth noting that Wiers *et al*. [18] and Eberl *et al*. [19] had a one-year follow-up period, whereas Schoenmakers *et al*. [17] had a three-month follow-up period. The different follow-up timing allows only a partial comparison of the above-mentioned proportions. If similar proportions at one-year of follow-up were also observed in Schoenmakers *et al*. [17] then the minimum number of cases would decrease to 10*2/0.47 ≅ 43.

An additional sensitivity power analysis was carried out for a simple logistic regression analysis on the binary outcome variable separately for the two cognitive bias re-training modules, to detect the minimal detectable effect in the present study as a function of significance level α, statistical power (1-β) and sample size. The null hypothesis (H_0_) stated that the probability (p_1_) of presenting a relapse event (Y = 1) in the active training condition (X = 1) equals the relapse rate in the placebo condition (X = 0) detected in previous studies (H_0_: p_1_ = p(Y = 1|X = 0) = p(Y = 1|X = 1)).

For the approach bias CBM training, p_1_ = 0.60, since about 60% of participants in the placebo condition relapsed [18,19], whereas for the attentional bias CBM training p_1_ = 0.18 [17]. The targeted value for the latter was 0.18; however, given the small sample size (n = 43) and almost 20% attrition rate in Schoenmakers *et al*. [17], similarly to the approach bias CBM training, a 60% relapse rate in the placebo condition is assumed.

The alternative hypothesis (H_1_) states that in the active condition the probability (p_2_) of presenting a relapse event is lower than in the placebo condition (H_1_: p_2_ = p(Y = 1|X = 1) < p_1_). Assuming a power of 0.80, a binomial distribution of Y and a one-tailed significance test, for n = 120 the projected odds-ratio or minimal detectable effect is 0.396 (critical z = −1.645) for both approach bias active versus placebo training and attentional bias active versus placebo training (G*Power 3.1, open-source software [53]).

## Discussion

The present paper describes the design of a double-blind RCT protocol to test the effectiveness of a computerised CBM intervention that targets alcohol-related maladaptive impulsive responses, which combines the attentional bias re-training and approach bias re-training in a 2×2 factorial design with the inclusion of motivational elements in each re-training session.

To the best of our knowledge, this is the first study to investigate the implementation of a combined CBM intervention alongside a targeted motivational intervention before each CBM session to sustain the training process in a clinical sample of outpatients. On a conceptual and clinical level, the combination of CBM with motivational elements specifically related to the contingent training context follows the dual-process account of addiction disorders, in which both the initial impulsive reactions to alcohol cues and the later in time, more reflective and deliberate processes can be targeted in intervention to bolster top-down control processes over impulses [8]. Weakening automatic appetitive processes by providing an alternative response option (training towards non-alcoholic stimuli) and simultaneously stressing the motivation (positive incentives) for more reflective and goal-directed behaviour is a double-pronged approach; both automatic and reflective processes are targeted. Furthermore, the motivation enhancement approach of the interview serves the purpose of levering participants’ commitment to and engagement in the training, which may well be perceived as monotonous and boring after a few sessions due to the inherent repetitiveness of the tasks.

One could argue that the MS should also have been experimentally manipulated to double-check its actual effects. Although that could have been experimentally optimal, it was not the main objective of the study. The leading focus was on the effects of the combination of two CBM interventions alongside the introduction of motivational elements. The MS was in fact conceived as a tool to optimise potential treatment effects of the CBM modules and to increase the acceptability and credibility of the CBM interventions, thus reducing attrition rates and improving the compliance to the training by subsuming the incentive value of long-term goal states [7,8].

A second strength of the present study bears the investigation of the combination of different CBM training with alcohol dependent outpatients. Up to now, the separate effects of CBM interventions have been mainly studied in samples of heavy drinkers [16,54] and residential inpatients [17-19], while the combined effects of multiple web-based CBM trainings are currently under investigation with a broader sample of at-risk and problematic drinkers [20]. Therefore, the second strength of the present study comes from the exploration of the potential effects of a CBM intervention in a different group of recipients who are also likely to benefit from the intervention. Alcohol dependent outpatients are usually characterised by cycles of relapses alternated with periods of abstinence, thus featuring their alcohol problems with a higher chronicity. When considering CBM modules as low-cost interventions that may potentially reduce the relapse rate and/or prolong the period of abstinence [17-19], therefore also prospectively deflating the number of patients that cyclically turn to addiction services to subside their relapse, the inclusion of CBM modules in the outpatient regimen of addiction interventions may be very cost-effective.

The third strength of the study lies in the adoption of a factorial design, which allows the detection of the main but also the incremental effects of combining two CBM interventions. Unfortunately, the main pitfall covers the lack of published results on the combination of different CBM paradigms, which did not permit the running of a proper *a priori* power analysis to set out the optimal sample size, as the effect of simultaneously targeting two cognitive biases at once is still unknown. The same applies to possible interaction effects between the two re-trainings, since as yet no published studies have tested, for instance, the effect of an approach bias re-training over the attentional bias or vice versa. These shortages could be considered as a drawback of the study in terms of power; however, they are also part of the main strengths, since the exploration of any interaction and/or additive effect of multiple CBM interventions is a key objective of the present study, and potentially fruitful for CBM research and clinical applications. Furthermore, a sensitivity power analysis for a multiple logistic regression makes it possible to at least have an ideal projection of the minimal detectable effect for the main effects of the two training modules. The identified values can then be used as a guideline when carrying out the data analysis of the present RCT and check for the actual sensitivity of the test in detecting the sought effect.

Last but not least, the RCT design includes the assessment of the effects of the CBM modules in the long run by measuring participants’ cognitive biases in a follow-up session. So far, only a few studies have investigated the long-term effects of CBM on cognitive biases, particularly for attentional bias [55]. Given the potential of CBM, more extended evaluations of training effects and of the relation between (long-lasting) cognitive bias change and primary outcome have been called for [55]. In summary, the present longitudinal assessment of attention and approach bias in alcohol outpatients can be valuable for both assessing its application in an important patient category and gaining deeper understanding of the working mechanisms of CBM.

A final remark goes to the generalizability of this RCT protocol, which is easily adaptable for other substances beyond alcohol and for diverse behavioural addictions, such as gambling and binge eating disorders. Clinical assessment measures and task stimuli can be customized for the targeted problems and/or disorder(s) while keeping unaltered the design of the experimental set-up and the CBM tasks. Better replicable RCT designs have been called for by a recent critical meta-analysis on the effectiveness of CBM interventions [56]. By extensively describing the RCT protocol separately, as done in the current paper, we hope to facilitate the cross-substance adaptation and reproducibility of CBM research findings.

## Trial status

The trial is currently in the active recruitment phase (started on July 2013).

### Consent

Written informed consent was obtained from the patient(s) for publication of this manuscript and accompanying images. A copy of the written consent is available for review by the Editor-in-Chief of this journal.

## Additional file

Additional file 1:
**Examples of task stimuli photographed in the six scenarios (three passive and three active): target and matched control stimuli for each alcohol category.**

## Abbreviations

AAT: Approach-Avoidance Task
ABPS: Amsterdam Beverage Picture Set
AUDIT: Alcohol Use Disorder Identification Test
BDI-II: Beck Depression Inventory-II
BIAT: Brief Implicit Association Test
CBM: Cognitive Bias Modification
MAC2-A: Motivation to treatment questionnaire (alcohol version)
MS: Motivational support
OCDS: Obsessive Compulsive Drinking Scale
RCT: Randomised controlled trial
RSES: Rosenberg Self-Esteem Scale
RT: Response time
SD: Standard deviation
STAI-Y: State-Trait Anxiety Inventory-Form Y
VPT: Visual Probe Task
