# Supplementary material for: Combining cognitive bias modification training with motivational support in alcohol dependent outpatients: study protocol for a randomised controlled trial
Source: Trials. 2015 Feb 26;16:63. doi: 10.1186/s13063-015-0576-6 (PMC4347655; doi:10.1186/s13063-015-0576-6)
Supplement: Additional file 1: — Examples of task stimuli photographed in the six scenarios (three passive and three active): target and matched control stimuli for each alcohol category. [file 13063_2015_576_MOESM1_ESM.docx]

**Table S1** Examples of task stimuli photographed in the six scenarios (three passive and three active): target and matched control stimuli for each alcohol category.

|  |  | Wine | | Beer | | Spirits | |
| --- | --- | --- | --- | --- | --- | --- | --- |
| Context | Scenario | Target | Control | Target | Control | Target | Control |
| Passive | Drink only | 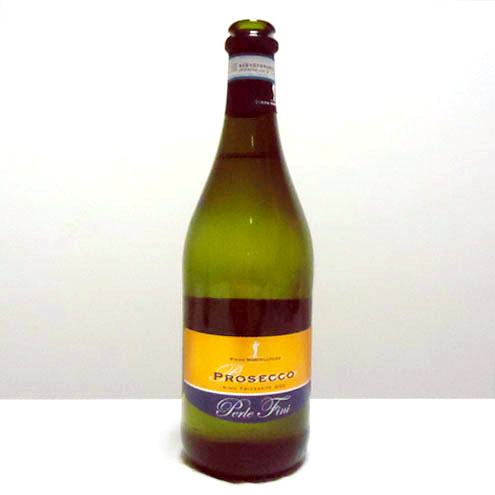 | 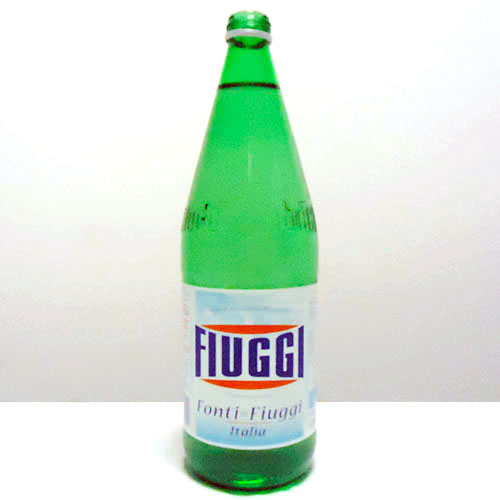 | 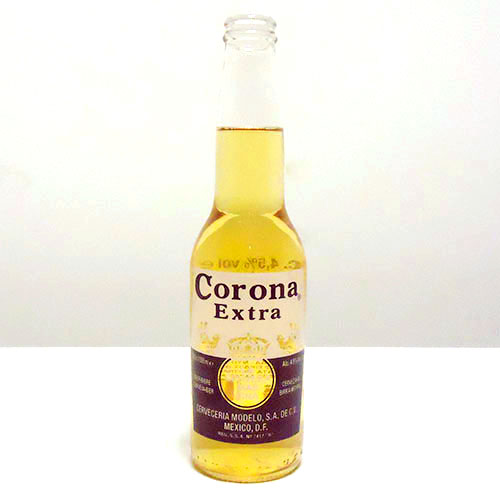 | 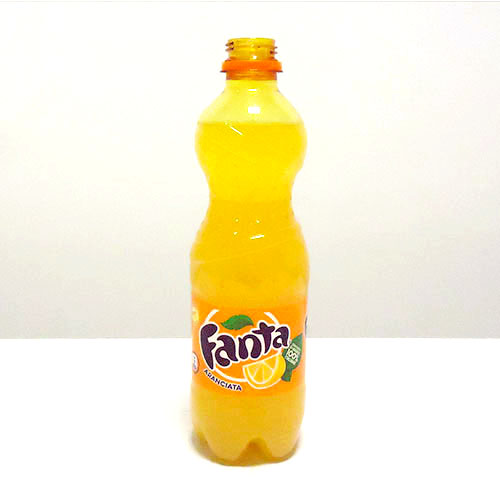 | 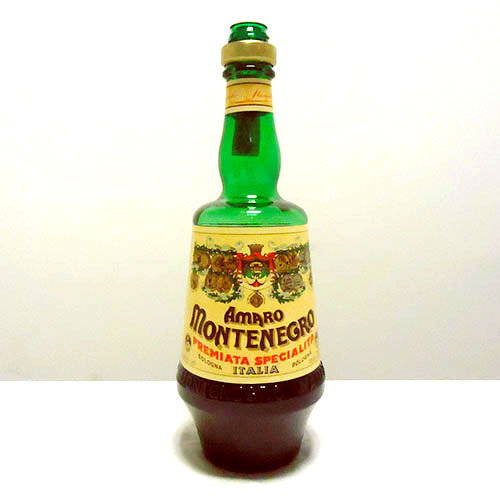 | 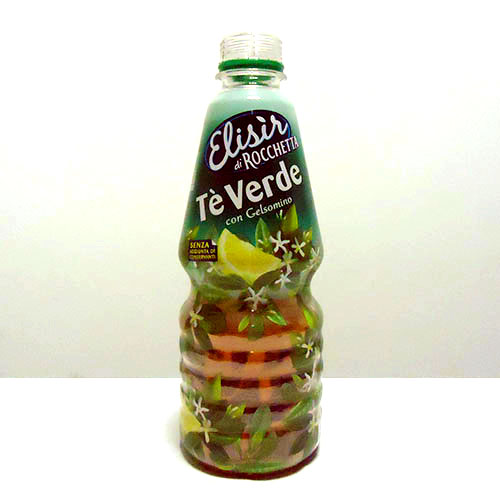 |
|  | Drink with empty glass | 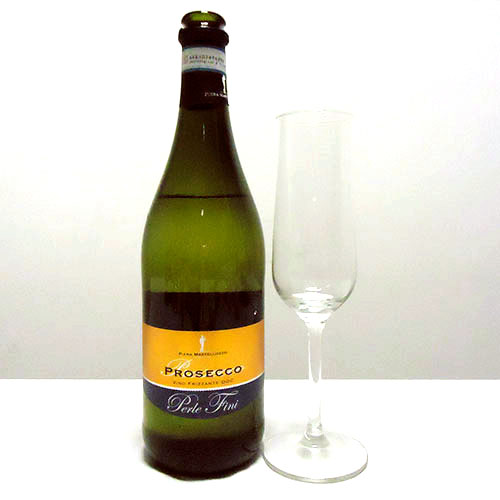 | 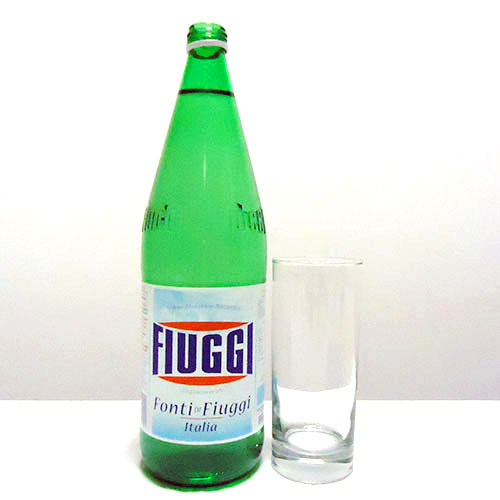 | 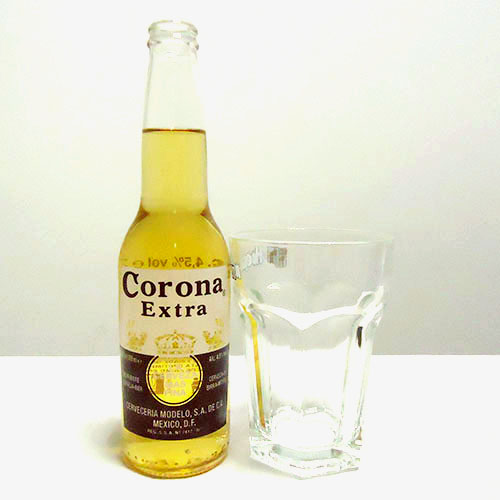 | 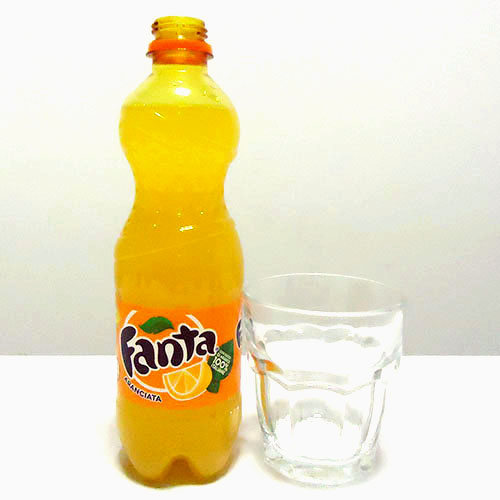 | 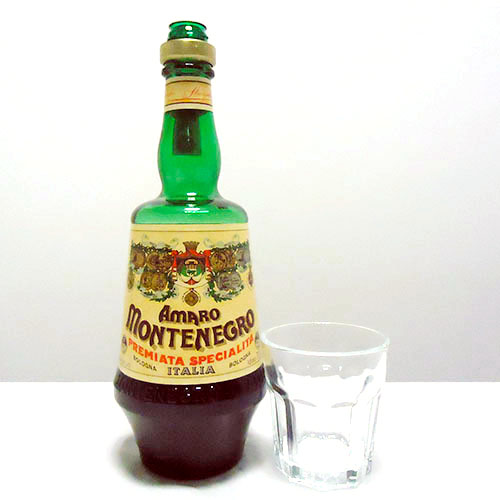 | 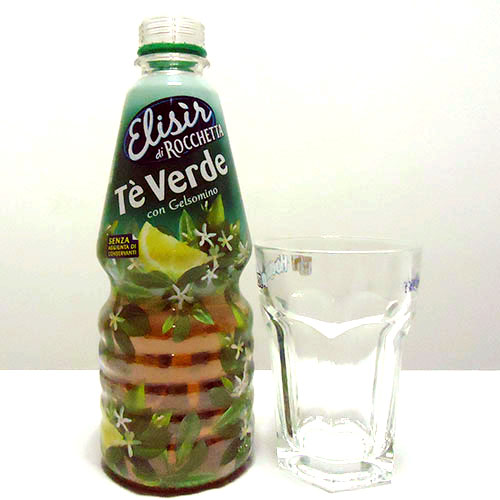 |
|  | Drink with full glass | 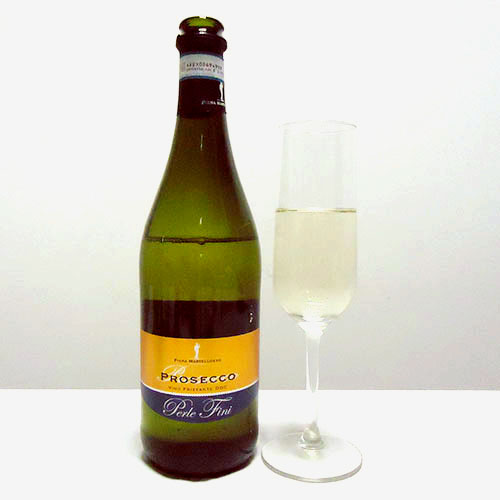 | 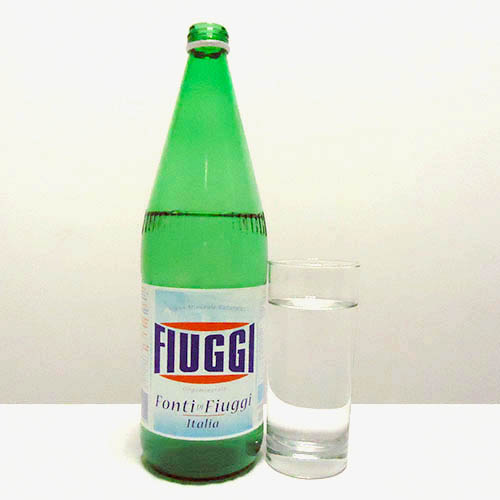 | 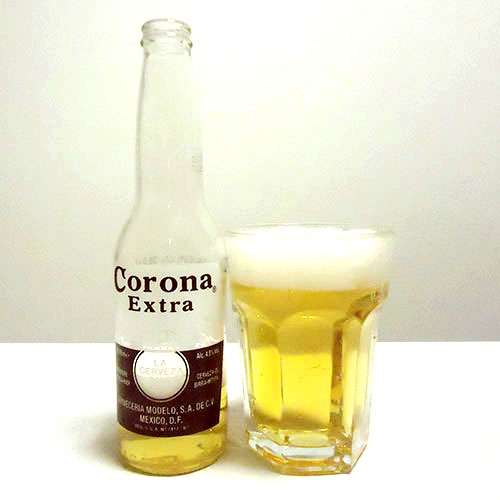 | 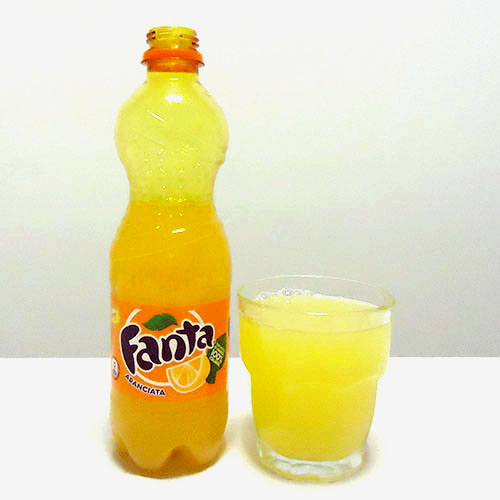 | 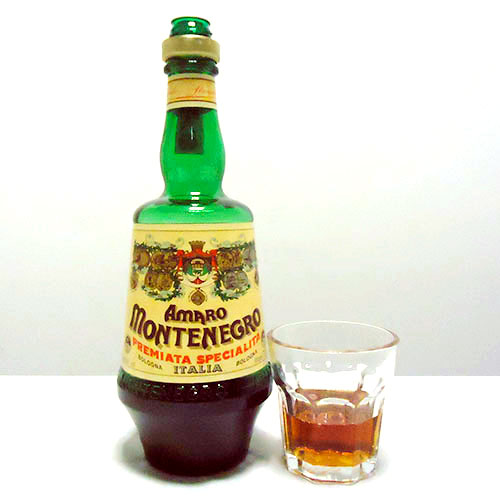 | 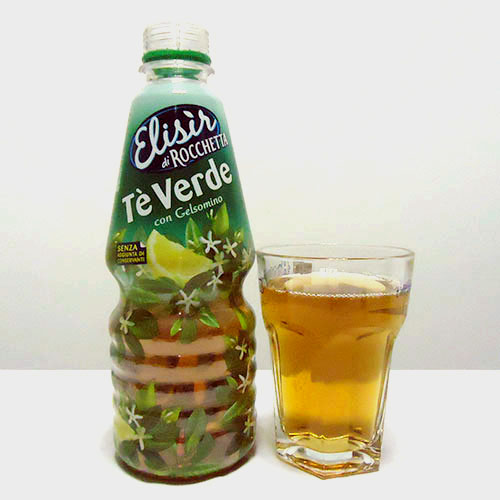 |
| Active | Serving | 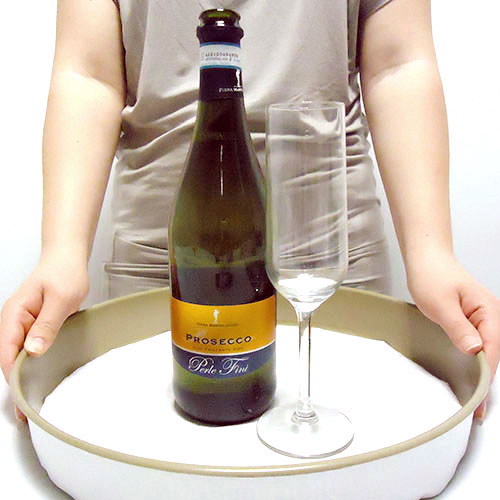 | 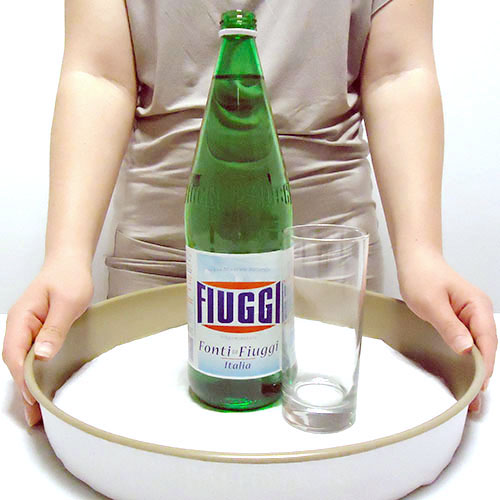 | 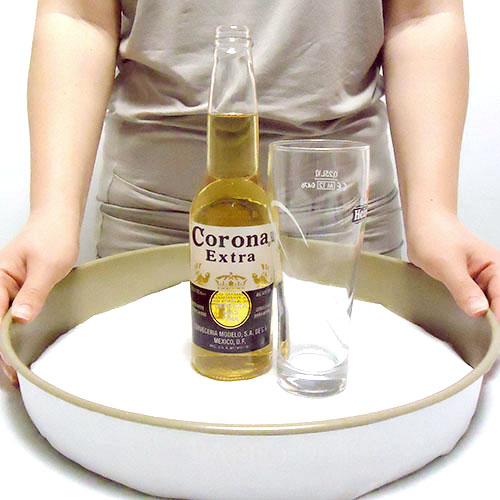 | 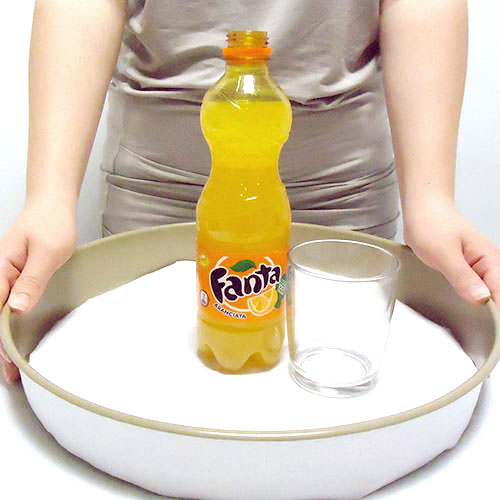 | 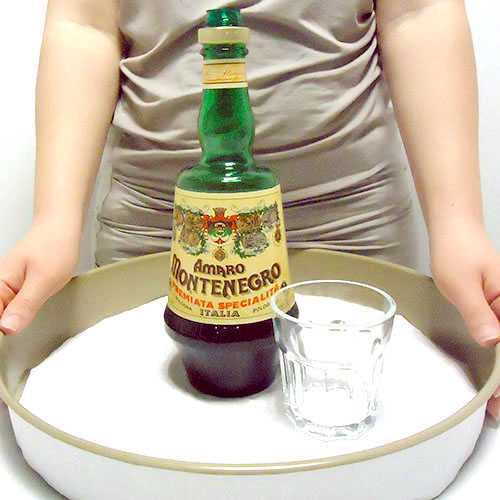 | 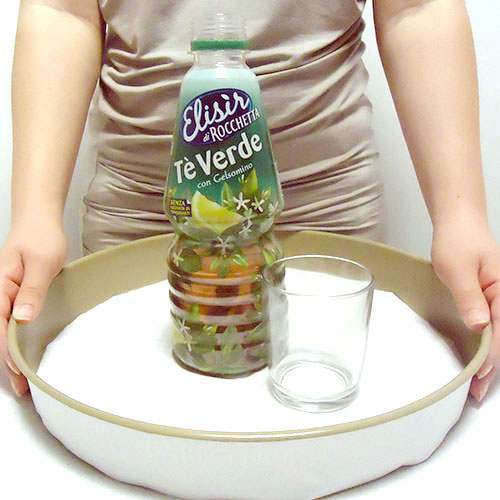 |
|  | Opening | 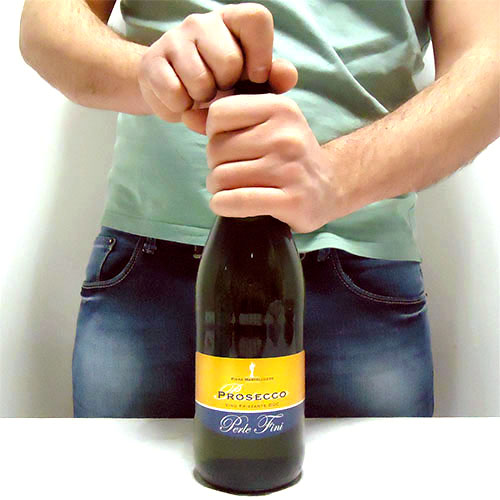 | 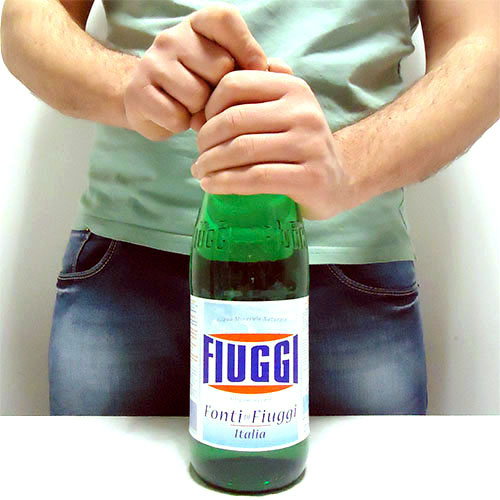 | 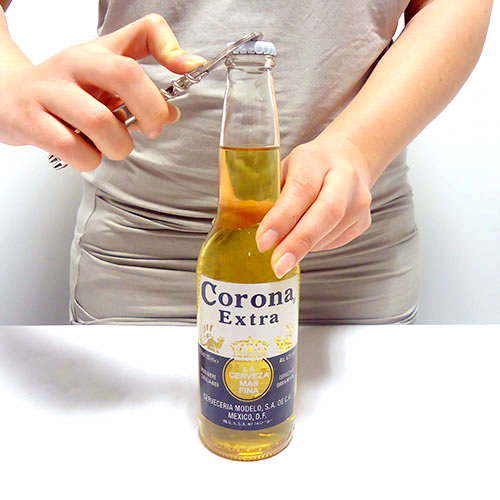 | 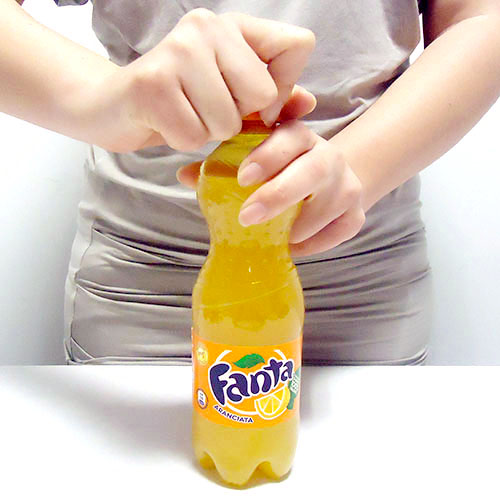 | 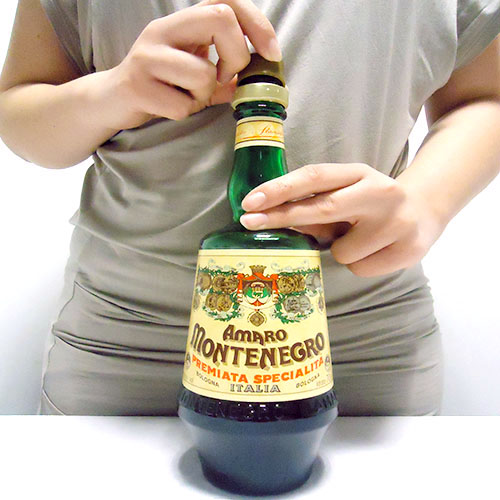 | 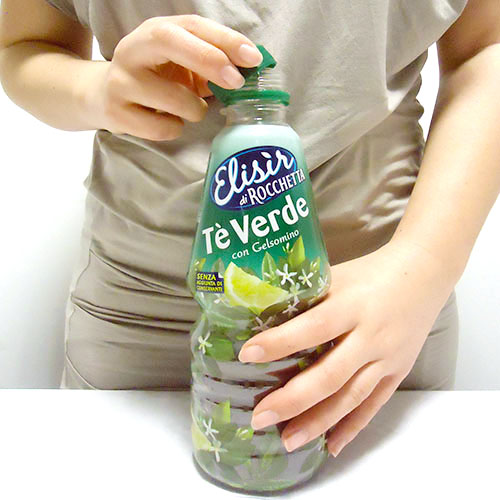 |
|  | Drinking | 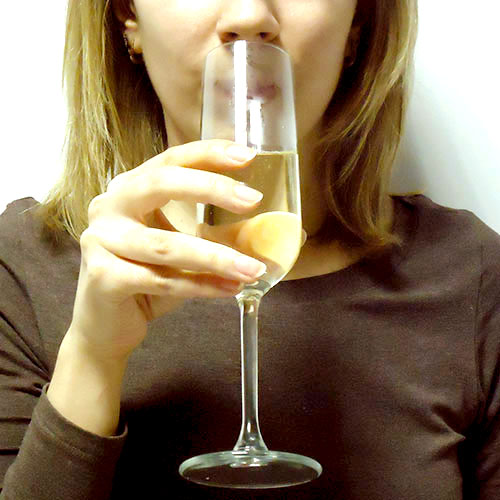 | 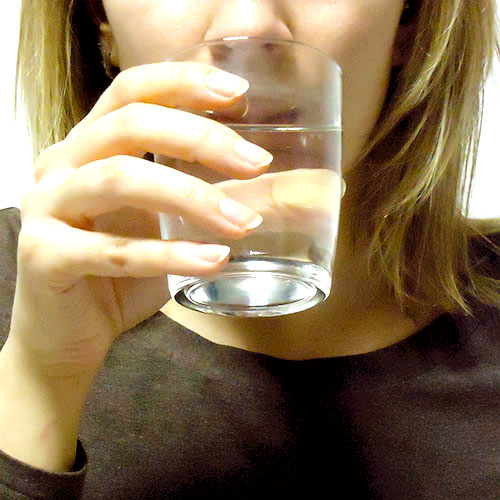 | 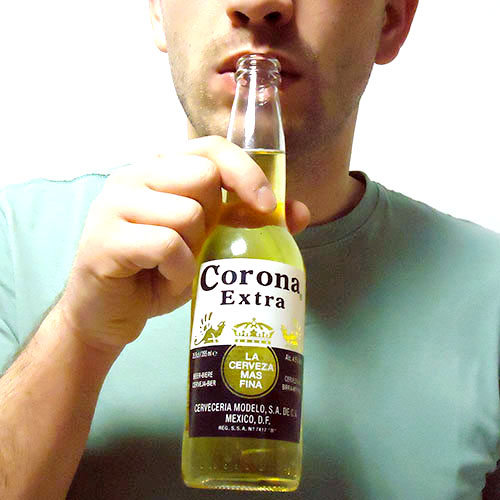 | 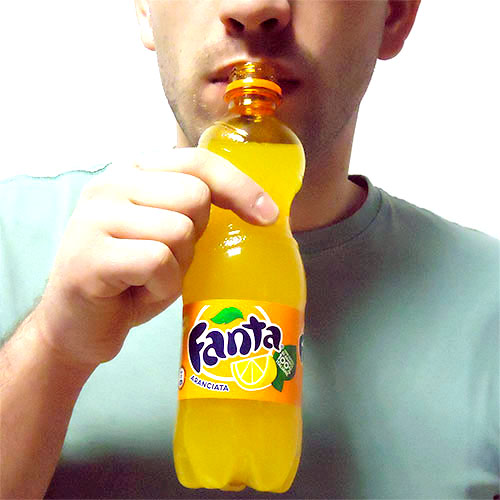 | 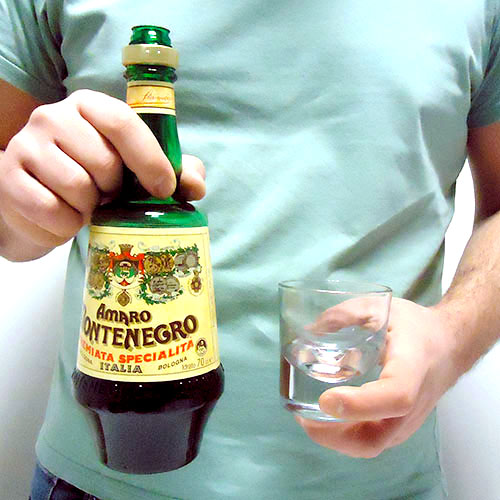 | 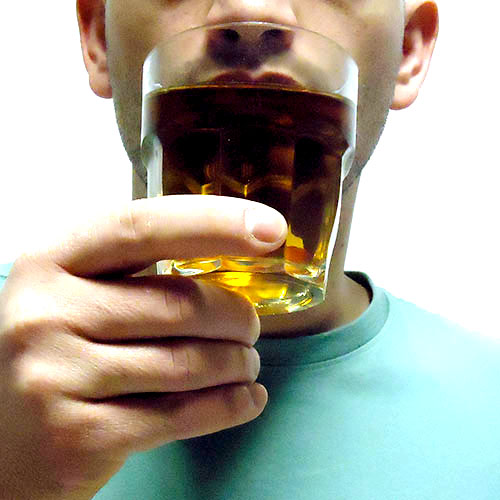 |
